# Therapeutic potential of human induced pluripotent stem cells and renal progenitor cells in experimental chronic kidney disease

**DOI:** 10.1186/s13287-020-02060-4

**Published:** 2020-12-09

**Authors:** Patrícia de Carvalho Ribeiro, Fernando Henrique Lojudice, Ida Maria Maximina Fernandes-Charpiot, Maria Alice Sperto Ferreira Baptista, Stanley de Almeida Araújo, Gloria Elisa Florido Mendes, Mari Cleide Sogayar, Mario Abbud-Filho, Heloisa Cristina Caldas

**Affiliations:** 1 Laboratory of Immunology and Experimental Transplantation (LITEX), Department of Medicine, FAMERP Medical School, Sao Jose do Rio Preto, SP, Brazil; 2 Cell and Molecular Therapy Center (NUCEL), School of Medicine, University of São Paulo, São Paulo, SP Brazil; 3 Kidney Transplant Unit, Hospital de Base, FAMERP/FUNFARME, Sao Jose do Rio Preto, SP Brazil; 4 Centro de Microscopia Eletrônica, Federal University of Minas Gerais, Belo Horizonte, Brazil; 5 Instituto de Nefropatologia, Belo Horizonte, Minas Gerais State Brazil; 6 Biochemistry Department, Chemistry Institute, University of São Paulo, São Paulo, SP Brazil

**Keywords:** Cell- and tissue-based therapy, Chronic kidney disease, Pluripotent stem cells, Stem cells

## Abstract

**Background:**

Chronic kidney disease (CKD) is a global public health problem. Cell therapy using pluripotent stem cells represents an attractive therapeutic approach for the treatment of CKD.

**Methods:**

We transplanted mitomycin C (MMC)-treated human induced pluripotent stem cells (hiPSCs) and renal progenitor cells (RPCs) into a CKD rat model system. The RPC and hiPSC cells were characterized by immunofluorescence and qRT-PCR. Untreated 5/6 nephrectomized rats were compared to CKD animals receiving the same amount of MMC-treated hiPSCs or RPCs. Renal function, histology, and immunohistochemistry were evaluated 45 days post-surgery.

**Results:**

We successfully generated hiPSCs from peripheral blood and differentiated them into RPCs expressing renal progenitor genes (PAX2, WT1, SIX2, and SALL1) and podocyte-related genes (SYNPO, NPHS1). RPCs also exhibited reduced OCT4 expression, confirming the loss of pluripotency. After cell transplantation into CKD rats, the body weight change was significantly increased in both hiPSC and RPC groups, in comparison with the control group. Creatinine clearance (CCr) was preserved only in the hiPSC group. Similarly, the number of macrophages in the kidneys of the hiPSC group reached a statistically significant reduction, when compared to control rats. Both treatments reduced positive staining for the marker α-smooth muscle actin. Histological features showed decreased tubulointerstitial damage (interstitial fibrosis and tubular atrophy) as well as a reduction in glomerulosclerosis in both iPSC and RPC groups.

**Conclusions:**

In conclusion, we describe that both MMC-treated hiPSCs and RPCs exert beneficial effects in attenuating CKD progression. Both cell types were equally efficient to reduce histological damage and weight loss caused by CKD. hiPSCs seem to be more efficient than RPCs, possibly due to a paracrine effect triggered by hiPSCs. These results demonstrate that the use of MMC-treated hiPSCs and RPCs improves clinical and histological CKD parameters, avoided tumor formation, and therefore may be a promising cell therapy strategy for CKD.

**Graphical abstract:**

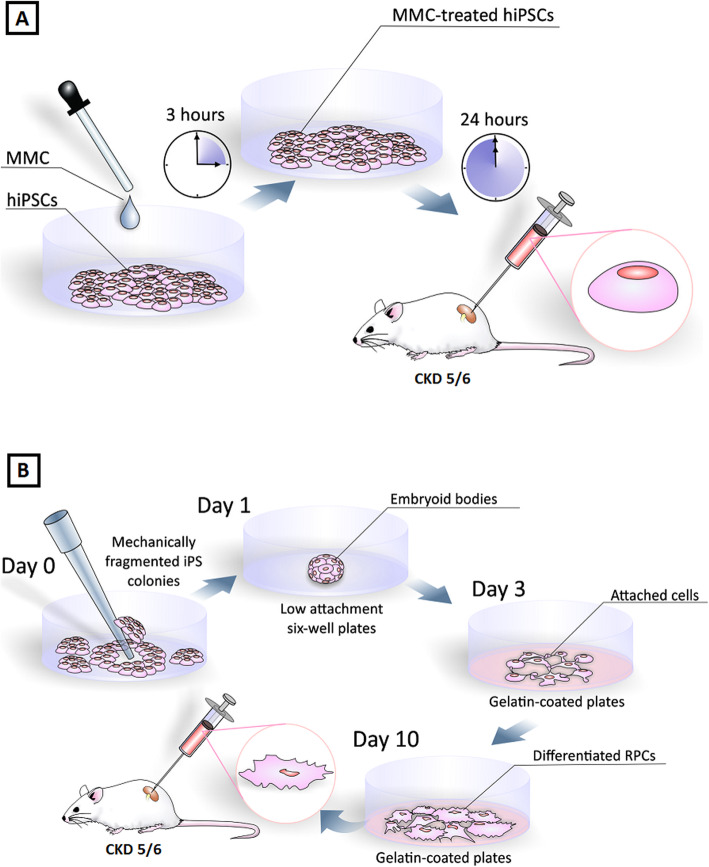

**Supplementary Information:**

The online version contains supplementary material available at 10.1186/s13287-020-02060-4.

## Background

The diagnosis of kidney diseases has improved over the years and is followed by a continuous increase in public and health professional awareness of chronic kidney disease (CKD). In line with this, the economic burden for National Health Systems worldwide is growing, with some countries spending more than half of their budget on renal replacement therapy for the 2% of CKD patients that evolve to kidney failure. Additionally, the excess of associated comorbidities significantly increases the whole cost relative to CKD treatment [1]. In such a scenario, the development of new alternative treatments, in addition to dialysis and transplantation, is urgently needed.

Cell therapy offers the use of pluripotent stem cells as an attractive therapeutic approach for the treatment of CKD in its different stages. Early stages of CKD may be more responsive to this type of therapy [2]. In this context, we have previously demonstrated that the injection of bone marrow-derived mesenchymal stem cells (BM-MSCs) from healthy rats in an experimental model of CKD had beneficial effects [3]. However, a major drawback of using BM-MSCs derived from uremic patients is that those cells did not convey functional protection, suggesting that autologous BM-MSCs are not suitable for CKD treatment [4]. Thus, the use of induced pluripotent stem cells (iPSCs) and renal progenitor cells (RPCs) may be a more efficient approach.

Induced pluripotent stem cells constitute a newly defined stem cell type with similar properties to those displayed by embryonic stem cells (ESCs), in terms of self-renewal and differentiation [5]. Additionally, iPSCs have a wide differentiation capacity without raising ethical conflicts, such as those observed with ESCs. However, because of the iPSC pluripotent nature, tumor formation has been reported after cell transplantation, restricting their use (in an immature stage) for cell therapy [3, 6]. Hence, blocking the proliferative ability of iPSCs with antimitotic agents, such as mitomycin C (MMC) and/or differentiating them into RPCs might be a safer strategy for CKD treatment [7–9].

In the present study, we sought the effects of hiPSCs and RPCs on the experimental CKD. To that end, we first generated hiPSCs and differentiated them into RPCs, and then we tested whether both cell types could be effective as potential therapeutic agents in a 5/6 nephrectomy model.

## Methods

### Reprogramming of PBMCs into pluripotent stem cells

Using the protocol described by Okita et al. [10], we derived hiPSCs from human peripheral blood mononuclear cells (PBMCs). Episomal vectors (pCE-hOct3/4, pCE-hSK, pCE-hUL, pCE-mp53DD, and pCXB-EBNA1) obtained from the Addgene plasmid repository were used for reprogramming.

PBMCs were collected from a healthy donor in the presence of anticoagulant agents and then purified by density gradient centrifugation with Ficoll-Paque Plus (GE Healthcare, density 1077 g/L) according to the manufacturer’s instructions. These isolated cells were cultured in X-VIVO-10 medium (Lonza) containing 100 ng/mL Erythropoietin (PeproTech), 10 ng/mL IL-3 (PeproTech), 50 ng/mL IGF-1, and 100 ng/mL stem cell factor (SCF; R&D Systems) for hematopoietic progenitor expansion. A mixture containing 1 μg of each plasmid was used for nucleofection of 1 × 10^6^ hematopoietic progenitors using the Nucleofector 4D (Lonza) device with a P3 Nucleofector kit according to the manufacturer’s instructions. ReproTeSR medium was added 48 h and 96 h after nucleofection and then changed every 48 h until reprogrammed colonies appeared (20–30 days after nucleofection). The colonies were manually passed and cultured in Matrigel^R^-treated plates in mTeSR-1 medium (StemCell Tech, Vancouver, BC, Canada).

To reduce the cell proliferation rate and the ability of hiPSCs to generate tumors, these cells were treated with mitomycin C (MMC), a chemotherapeutic agent which is capable of arresting cell proliferation [7, 8], 24 h before transplantation. MMC (10 μg/mL) was added to the culture medium for 3 h, before fresh medium addition [11].

### hiPSC differentiation into renal progenitor cells

The differentiation protocol followed was previously described by Song et al. [9]. The hiPSC colonies were mechanically fragmented into pieces of similar size. The fragments obtained were then transferred to ultra-low attachment six-well plates (Corning Costar) and maintained in culture for 72 h in differentiation medium, which consisted of DMEM F-12 (50/50%) (Gibco), 2.5% fetal bovine serum (FBS; Gibco), 0.1 mM non-essential amino acids, 0.1 mM beta-mercaptoethanol (Gibco), and 1% antibiotic-antimycotic (Gibco), and were supplemented with the following nephrogenic factors: 0.1 μM retinoic acid (RA) (Tocris), 10 ng/mL activin A (PeproTech), and 15 ng/mL BMP7 (PrepoTech).

After 72 h in ultra-low attachment plates, the embryoid bodies (EBs) generated were transferred to culture plates coated with 0.1% gelatin (Sigma), with cells adhering and proliferating in differentiation medium for another 7–8 days. The cells were then seeded using the basal medium, without nephrogenic factors, until their characterization. During the entire culture process, the medium was changed every other day and cells were expanded with trypsin.

### Immunofluorescence staining

For immunofluorescence staining, hiPSC colonies and RPCs were fixed and permeabilized using 4% paraformaldehyde and 1% Triton X-100. Samples were blocked with 5% bovine serum albumin (BSA) for 1 h at room temperature. hiPSC colonies were stained overnight with unconjugated anti-OCT4A Rabbit mAb (Cell Signaling Technology, 1:200), anti-Nanog (Cell Signaling Technology, 1:200), anti-PAX2 (Abcam, 1:100), WT-1 (Abcam, 1:100), anti-Nephrin (Thermo Scientific, 10 μg/mL), and anti-Sinaptopodin (Thermo Scientific, 20 μg/mL). The RPCs were stained overnight with primary antibodies to PAX2 (Abcam, 1:100), WT-1 (Abcam, 1:100), Nephrin (Thermo Scientific, 10 μg/mL), and Sinaptopodin (Thermo Scientific, 20 μg/mL). On the next day, samples were stained with the Alexa Fluor 488 secondary antibody (Life Technologies, Carlsbad, CA, USA, 1:1000). Subsequently, slides were mounted with Vecta Shield Antifade Mounting Medium (Vector Laboratories, Burlingame, CA, USA) containing DAPI, and images were acquired using the EVOS FL Imaging System (Thermo Fisher Scientific, Waltham, MA, USA).

### RNA isolation from culture cells and quantitative real-time PCR (qRT-PCR)

RNA from erythroblasts, hiPSCs, EBs, and RPCs was extracted using the Illustra RNAspin Mini Kit (GE Healthcare) method, following the manufacturer’s protocol. The RNA was transcribed into complementary DNA (cDNA) using 1 μg of total RNA and the High-Capacity cDNA Reverse Transcription kit (Applied Biosystems), following the manufacturer’s instructions.

Quantitative real-time PCR was carried out using TaqMan Gene expression Master Mix (Applied Biosystems). Primers to the following genes were used for hiPSC and RPC expression analysis: PAX2, SIX2, WT1, SALL1, NPHS1, SYNPO, OCT4, NANOG, ACTB, GAPDH, and HMBS. The primers used in the amplification reactions are shown in the additional file (see Additional Table 1). The threshold cycle (Ct) values were measured in triplicate and normalized against the endogenous controls (ACTB, GAPDH, and HMBS). Erythroblasts or human kidney tissue served as reference controls.

### In vivo experiments

This study was carried out in strict accordance with the recommendations of the Guide for the Care and Use of Laboratory Animals of the National Institutes of Health. The experimental protocol was previously approved by the Ethics Committee of FAMERP Medical School (São Jose do Rio Preto, Brazil—permit number: 001-001788/2017). All surgeries were performed under anesthesia, and all efforts were made to minimize animal suffering. Overdose anesthetic (sodium pentobarbital) was the euthanasia method in this study.

Thirty-two male Wistar rats (250–300 g) were maintained under a 12-h light/dark cycle, with food and water available ad libitum. The 5/6 reduction of renal mass was performed in accordance with previously described techniques [12]. Briefly, male rats were anesthetized with xylazine (10 mg/kg) and ketamine hydrochloride (50 mg/kg). Trichotomy and antisepsis of the ventral abdominal area were conducted, and animals were positioned on horizontal dorsal decubitus. Subtotal renal ablation was carried out in a surgical procedure through a right nephrectomy and selective infarction of two thirds of the left kidney by the ligation of extrarenal branches of the left renal artery, remaining approximately one-sixth of the total kidney tissue mass without lesions.

### Experimental design

The rats were divided into the following groups: CKD (*n* = 8), which received only cell culture medium after the surgery; RPC (*n* = 8), which received 1 × 10^6^ RPCs (*n* = 8); hiPSC (*n* = 8), which received 1 × 10^6^ MMC-treated hiPSCs; and sham-operated animals (*n* = 8).

Immediately after mass reduction and right nephrectomy, hiPSCs, RPCs, or culture medium were injected into the renal parenchyma, specifically, between the infarcted and the healthy areas. The abdominal cavity was then sutured and all animals were observed for 45 days.

### Renal function analysis

To collect 24-h urine, all animals were weighed and allocated to metabolic cages (Tecniplast, Buguggiate, VA, Italy) at baseline and 45 days after surgery. Also, during the surgery and immediately before the euthanasia, blood was collected from the cava vein. Renal function was evaluated by the dosages of serum creatinine (SCr), creatinine clearance (CCr), rate of decline of CCr (RCCr), and 24-h proteinuria (PT-24h). The rate of decline in the CCr (RCCr; mL/min/day) was used as a measure of CKD progression. After 45 days of surgery, all animals were euthanized, and the kidneys were processed for histological and immunohistochemistry analysis.

A colorimetric assay (spectrophotometer BTS 310; Biosystems S.A., Barcelona, Spain) was used to measure plasma and urine creatinine SCr and urine protein concentrations. Creatinine clearance and the rate of decline of clearance were calculated from the dosages of serum and urinary creatinine levels. Blood pressure was evaluated by an indirect tail-cuff method (Insight LTDA, Ribeirão Preto, SP, Brazil), and the average of three measurements was used for analysis.

### Histological and immunohistochemical analyses

For histological evaluation, the renal tissue was fixed in formalin and embedded in paraffin. The sections were stained with hematoxylin and eosin (HE) and Masson’s trichrome. A semi-quantitative score was derived for each sample, to determine the extent of tubule-interstitial changes, as previously described [13]. The interstitial fibrosis and tubular atrophy (IFTA) score was obtained according to the appropriate proportion of tissue affected (0 to 100%). For glomerulosclerosis analysis, the number of affected glomeruli was counted and divided by the total glomeruli number. All sections were evaluated by a blind observer.

Immunohistochemical (IHC) staining was performed as previously described [14]. The tissue sections were incubated using the following primary antibodies: CD68 (Serotec, MCA1957GA, 1:250), anti-human nucleoli antigen antibody [NM95] (Abcam, ab190710, 1:500), and α-smooth muscle actin (α-SMA) (Dako, clone 1A4, cat. M0851, 1:100). All primary antibodies were incubated overnight at 4 °C and an IHC detection kit was used (Abcam, Mouse and Rabbit Specific HRP/DAB (ABC) Detection IHC kit (ab64264)). For evaluation of immunoperoxidase staining for α-SMA, each grid field was semi-quantitatively graded and the mean score per kidney was calculated. Each score reflected mainly changes in the extent, rather than the intensity, of staining and depended upon the percentage of grid field showing positive staining: 0 = absent or less than 5%, I = 5–25%, II = 25–50%, III = 50–75%, and IV > 75%. The number of CD68-positive cells in each section was calculated by counting the number of positive cells in 30 sequential grid fields (0.245 mm^2^) from the renal cortex [14].

### Statistical analysis

Analyses were performed using StatsDirect version 3.0 software (*StatsDirect* Ltd., Cheshire, UK), with the critical level set at *p* < 0.05. Data were expressed as mean ± standard deviation. Comparisons among multiple groups were performed using analysis of variance (ANOVA). When *p* values were significant, differences between the groups were specified using Tukey’s post hoc multiple comparison test. When comparing data, the two-sided Student’s *t* test and Mann-Whitney *U* tests were used.

## Results

### Generation and characterization of hiPSCs

**Fig. 1:**
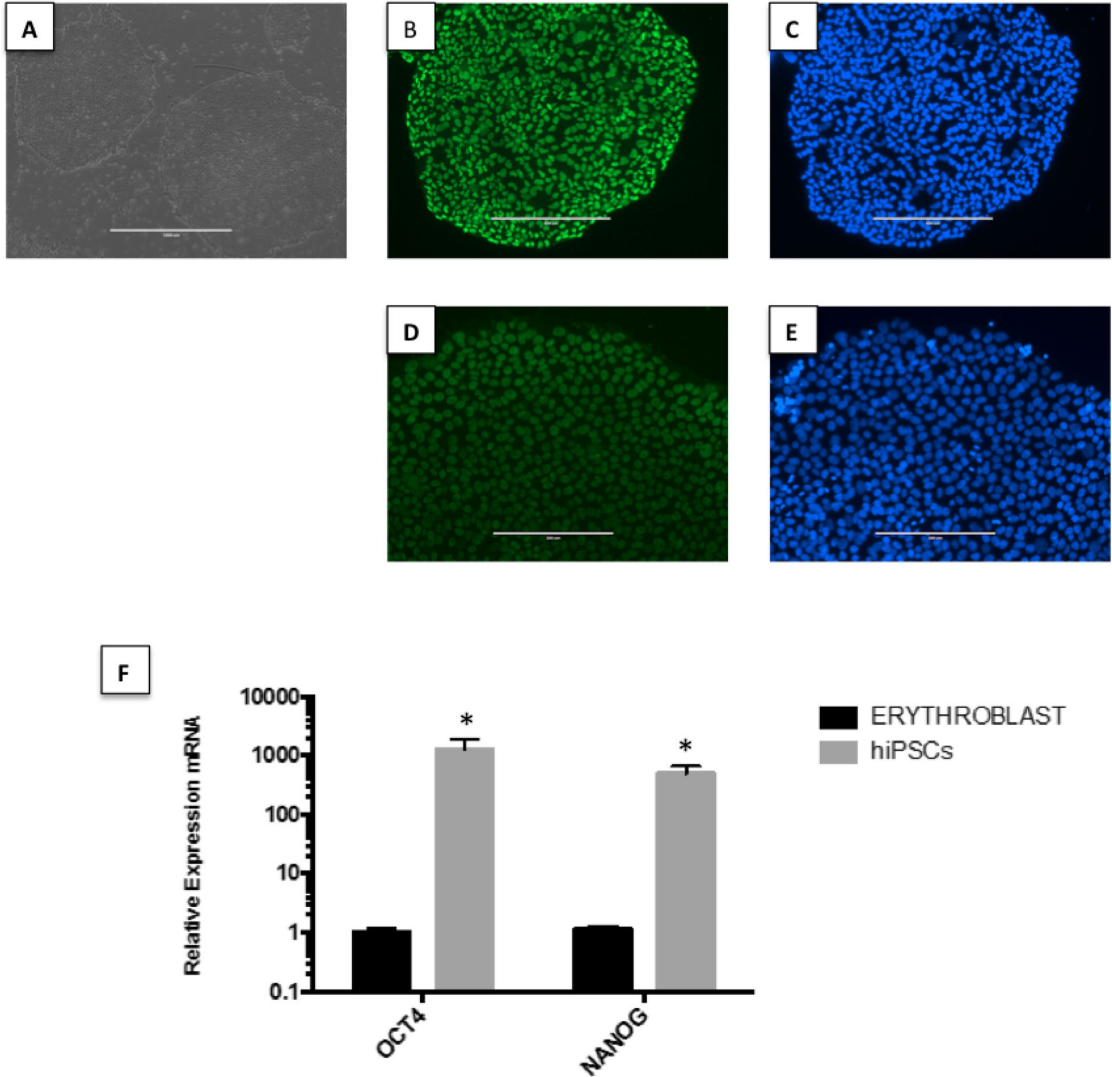
Characterization of peripheral blood-derived hiPSCs. **a** Typical morphology of undifferentiated hiPSC colonies. The colonies grown over Matrigel have a homogeneous shape, smooth and regular edges, and positive staining for **b** OCT4, **d** NANOG, and **c**, **e** DAPI staining for the same colonies of **b** and **d**, respectively. Scale bar 200 μm. **f** Gene expression levels in samples collected before (erythroblasts) and after (hiPSCs) reprogramming, generated by referencing each gene to HMBS expression levels as an internal control. Healthy erythroblast mRNA was used as the comparative sample. *p value < 0.05 vs. erythroblast

Approximately 20–25 days after the nucleofection process, it was possible to observe the formation of hiPSC colonies (Fig. 1). The cells showed pluripotent stem cell morphology, a high nucleus/cytoplasm ratio, refringent colonies, and a high proliferative rate in culture.

To further characterize the generated hiPSCs, we used immunofluorescence and qRT-PCR analysis. The colonies displayed positive staining for pluripotency markers, as well as a high expression of OCT4 and Nanog (Fig. 1), demonstrating their pluripotent nature.

### Differentiation of hiPSCs into RPCs

The hiPSC differentiation process consisted of a 10-day protocol, involving a combination of RA, activin A, and BMP7. The process began with the manual fragmentation of hiPSC colonies into pieces of similar sizes. Approximately 24 h later, it was possible to observe fragment aggregation, forming non-adherent cell clusters, i.e., embryoid bodies.

After adhesion onto gelatin-coated plates, in the continuous presence of the differentiation medium, cells began to undergo morphological changes. At early stages, cells appeared as single cells, but throughout the differentiation process, their morphology became increasingly similar to renal podocytes. In the final days of the differentiation process, it was possible to observe cytoplasmic extensions with an arborized appearance.

### hiPSC and RPC characterization

**Fig. 2:**
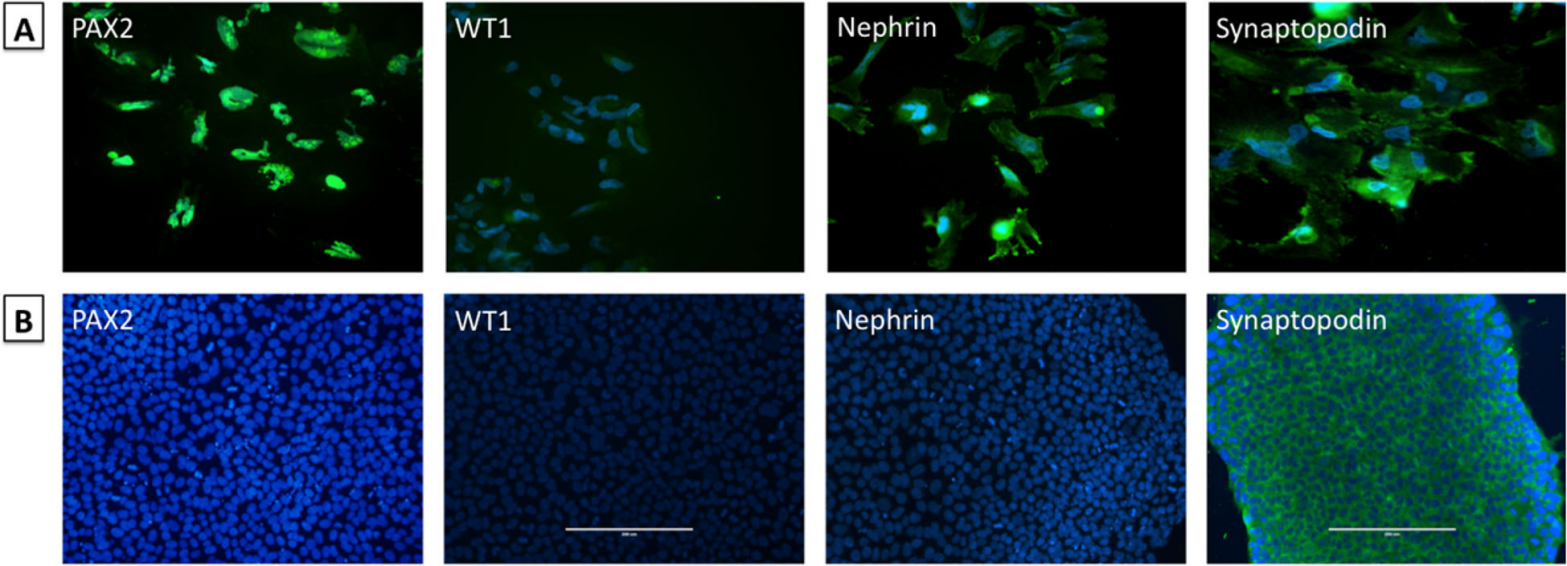
Immunofluorescence staining of cells during differentiation process of hiPSC into podocyte-like cells. **a** RPCs were positive for renal progenitor markers (PAX2 and WT1, nuclear and cytoplasmic, respectively) and renal podocyte markers (nephrin and synaptopodin, cytoplasmic) on day 12. Magnification × 40. **b** hiPSCs were negative for PAX2, WT1, and Nephrin, and positive for Synaptopodin on day 0. Magnification × 20

The hiPSCs and RPCs were characterized by immunofluorescence and qRT-PCR analysis. On day 0 of the differentiation process, the hiPSCs were negative for immunostaining with PAX2, WT1, and Nephrin, and positive for the Synaptopodin podocyte marker (Fig. 2). After 12 days, PAX2, WT-1, Nephrin, and Synaptopodin staining confirmed the successful differentiation of the hiPSCs into RPCs. The differentiated cells showed positive staining for PAX2 and WT1 (renal progenitor markers) and for Nephrin and Synaptopodin (podocyte markers), indicating their successful differentiation into renal podocyte progenitors (Fig. 2). A total of 97% of the differentiated cells presented a positive immunostaining for RPC markers.

**Fig. 3:**
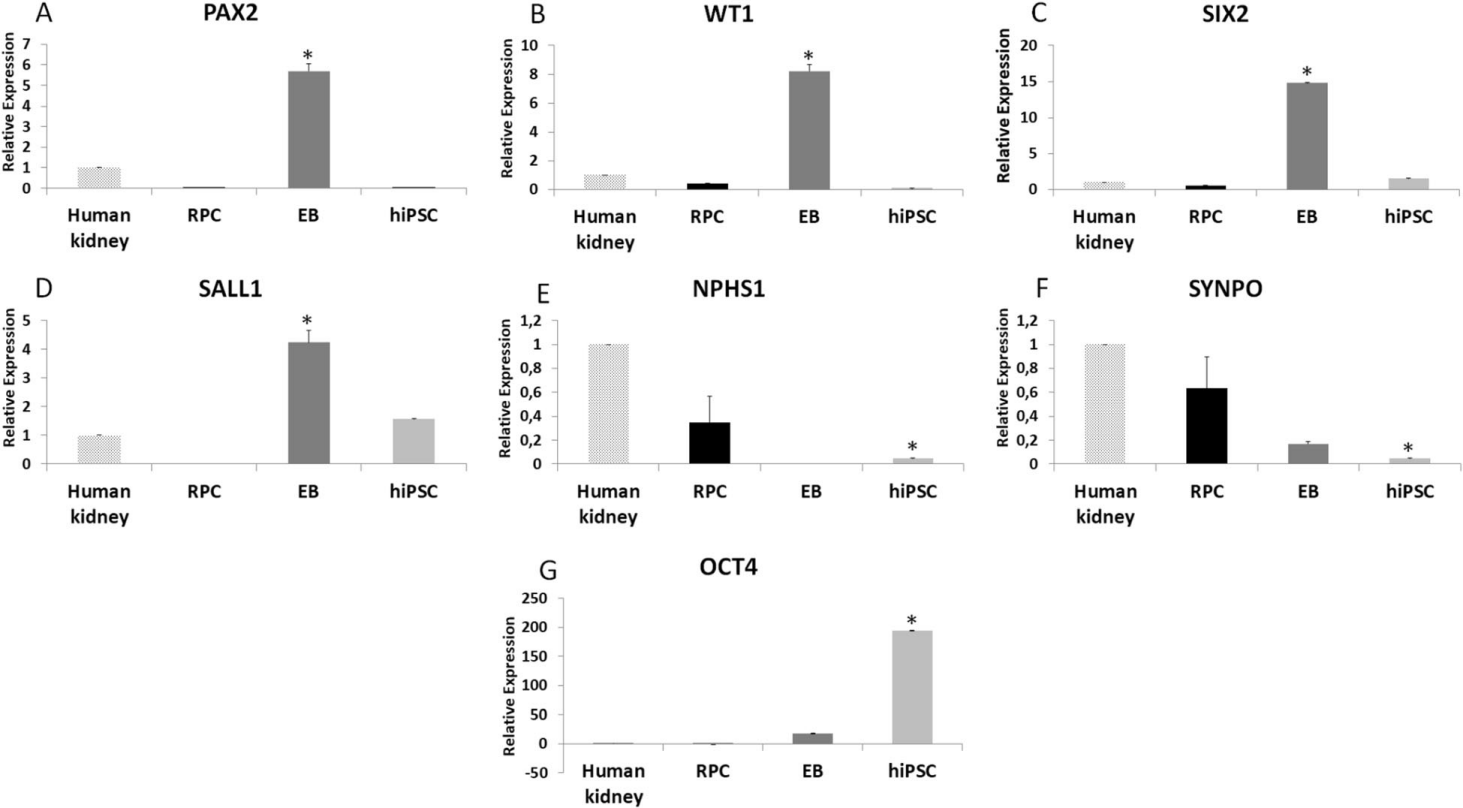
Quantitative real-time PCR for RPC (day 16), EB (day 3), and hiPSC (day 0). EBs showed an increased expression of **a** PAX2 (*p* = 0.001 vs. RPC, *p* = 0.002 vs. hiPSC), **b** WT1 (*p* = 0.03 vs. RPC, *p* = 0.002 vs. hiPSC), **c** SIX2 (*p* = 0.0001 vs. RPC, *p* = 0.0001 vs. hiPSC), and **d** SALL1 (*p* = 0.05 vs. RPC, *p* = 0.01 vs. hiPSC). hiPSCs showed a decreased expression of **e** NPHS1 (*p* = 0.001 vs. human kidney) and **f** SYNPO (*p* = 0.03 vs. RPC, *p* = 0.01 vs. EB, *p* = 0.0002 vs. human kidney) on day 0, whereas the expression of both **e** NPSH1 and **f** SYNPO increased in RPCs on day 16. The pluripotency marker gene **g** OCT4 had a decreased expression during the differentiation process (*p* = 0.04 hiPSC vs. EB, *p* = 0.02 hiPSC vs. RPC). *p value < 0.05

The qRT-PCR results, shown in Fig. 3, indicate the increased expression of renal progenitor genes (PAX2, WT1, SIX2, and SALL 1) during the initial phase of the differentiation process and increased expression of renal podocyte genes (NPHS1 and SYNPO) after 10 days of the differentiation process. When compared to the undifferentiated hiPSCs and/or RPCs, the EBs showed an increased expression of PAX2, WT1, SIX2, and SALL1. After the differentiation process, RPCs displayed increased expression of the NPHS1 and SYNPO genes, when compared to hiPSCs and EBs. hiPSCs showed a decreased expression of the NPHS1 and SYNPO genes. Decreased expression of OCT4 was also observed throughout the differentiation process (Fig. 3).

### Effect of hiPSCs and RPCs on clinical and histological parameters

As shown in Table 1, the body weight change was significantly lower in the CKD group, when compared to sham rats. In contrast, the body weight change in the treated groups was significantly increased when compared to the CKD group, similarly to the weight change observed in the sham group.

**Table 1:** Renal function parameter measurements at the end of the study (day 45)

Parameters	Groups			
	Sham	CKD	RPC	hiPSC
**Body weight change (g)**	61.6 ± 36.2^a^	− 23.6 ± 101^b,c^	56.7 ± 23	59.7 ± 14
**Mean arterial pressure (mmHg)**	127 ± 1	201 ± 23	172 ± 30	177 ± 37
**CCr (mL/min)**	1 ± 0.1	0.85 ± 0.1^d^	0.91 ± 0.5^e^	1.61 ± 0.5
**RCCr (mL/min/day)**	0.001 ± 0.003	0.01 ± 0.01	0.005 ± 0.01	0.002 ± 0.008
**SCr (mg/dL)**	0.6 ± 0.1^f^	1.06 ± 0.5	0.87 ± 0.24	0.85 ± 0.13
**PT24h (mg/24 h)**	10 ± 0.2^g^	44.5 ± 22.6	45.8 ± 30	36 ± 21

Results are presented as mean ± SD. *Abbreviations*: *SCr* serum creatinine, *RCCr* rate of decline of CCr, *PT24h* 24-h proteinuria, *CCr* creatinine clearance, *CKD* chronic kidney disease, *RPC* renal progenitor cell, *hiPSC* induced pluripotent stem cell. ^a^*p* < 0.05 vs. CKD; ^b,c^*p* < 0.05 vs. RPC and hiPSC; ^d^*p* < 0.05 vs. hiPSC; ^e^*p* < 0.05 vs. hiPSC, CKD; ^f^*p* < 0.01 vs. CKD; ^g^*p* < 0.01 vs. CKD vs. RPC vs. hiPSC

The mean arterial blood pressure was numerically reduced in the treated groups when compared to the CKD group (CKD = 201 ± 23.4 mmHg vs. RPC = 172 ± 30 mmHg vs. hiPSC = 177 ± 37 mmHg), but the difference did not reach statistical significance.

### Renal functional studies

Only the treatment with hiPSC significantly preserved the creatinine clearance, when compared with the CKD and RPC groups (CKD = 0.85 ± 0.1 mL/min vs. RPC = 0.91 ± 0.5 mL/min/day vs. hiPSC = 1.61 ± 0.5 mL/min/day, *p* < 0.05). Although not statistically significant, treatment with both RPCs and hiPSCs slowed the RCCr by 50% and 80%, respectively, when compared to CKD rats (CKD = 0.01 ± 0.01 mL/min/day vs. RPC = 0.005 ± 0.01 mL/min/day vs. hiPSC = 0.002 ± 0.008 mL/min/day, *p* = 0.4). SCr was partially reduced by both cell treatments, while PT-24h remained unchanged during the observation period (Table 1).

### Histological and immunohistochemical analyses

Histological analysis showed that kidneys from hiPSC and RPC groups exhibited less tubulointerstitial damage and less glomerulosclerosis, measured by lower IFTA scores, and better preservation of glomerular structures, when compared to the CKD group (Table 2).

**Table 2:** Histological changes and the effect of cell treatment in the 5/6 nephrectomized animals

	CKD	RPC	hiPSC
**IFTA**	44.9 ± 26^a,b^	15.8 ± 13.2	13.6 ± 13.1
**Glomerulosclerosis**	0.13 ± 0.13^c,d^	0.01 ± 0.01	0.008 ± 0.01

Data are expressed as means ± SD. *Abbreviation*: *IFTA* interstitial fibrosis and tubular atrophy. ^a^*p* = 0.003 vs. RPC; ^b^*p* = 0.01 vs. hiPSC; ^c^*p* = 0.01 vs. RPC; ^d^*p* = 0.01 vs. hiPSC

**Fig. 4:**
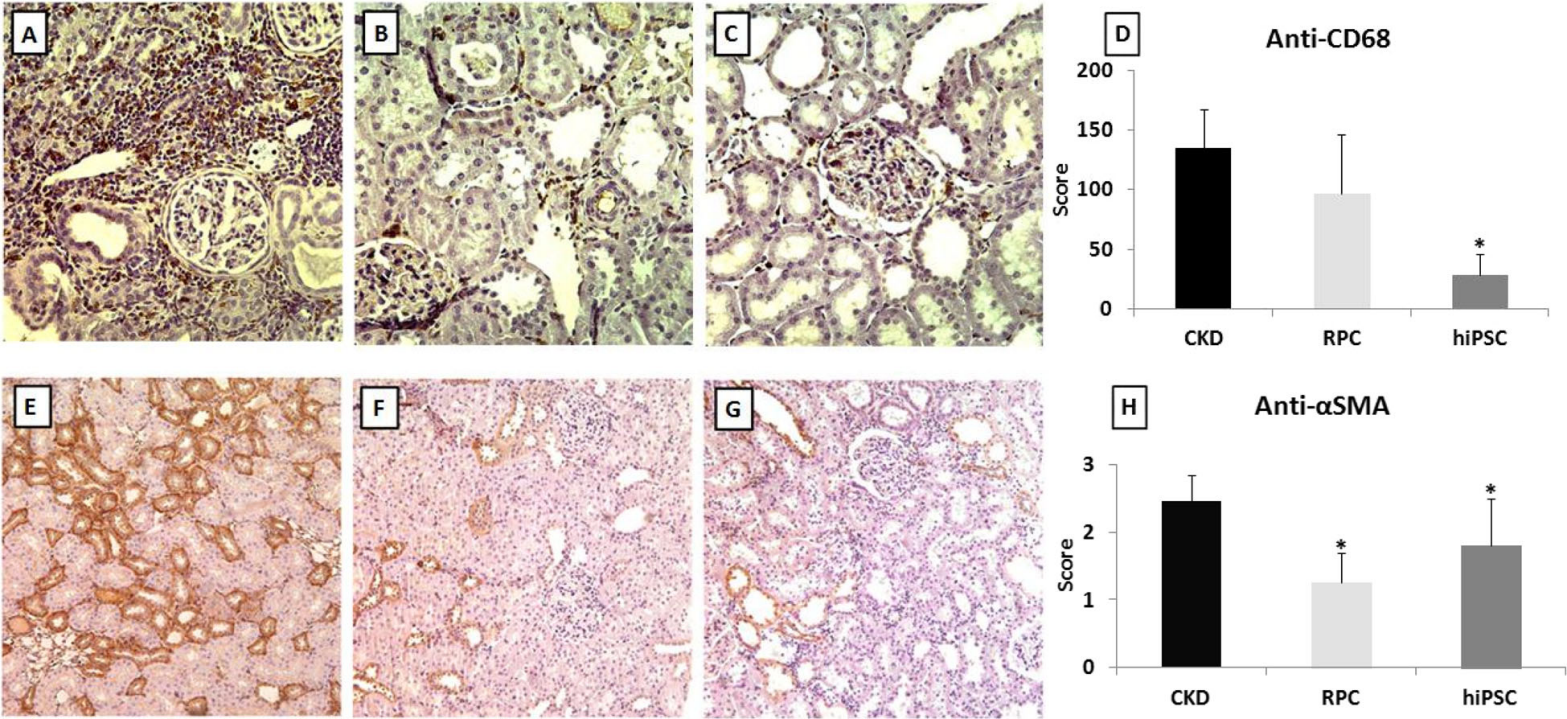
Immunohistochemistry and quantitative analysis for anti-CD68 and anti-α-SMA. Anti-CD68 for the **a** CKD group, **b** RPC group, and **c** hiPSC group, magnification of × 40. **d** Anti-CD68 quantitative analysis for all the three groups. Anti-α-SMA for the **e** CKD group, **f** RPC group, and **g** hiPSC group, magnification of × 40. **h** Anti-α-SMA quantitative analysis for all the three groups. *p value < 0.05 vs. CKD

The immunohistochemistry analysis showed that the hiPSC group had a reduced number of macrophages in renal tissue, as showed by a greater decrease in the positive staining for CD68, when compared to the CKD group (CKD = 135 ± 32 vs. hiPSC = 27.9 ± 18, *p* < 0.001). Both cell types significantly reduced the positive staining for α-SMA (CKD = 2.46 ± 0.3 vs. RPC = 1.3 ± 0.4 vs. hiPSC = 1.79 ± 0.7, *p* = 0.0006) suggesting a reduction in fibrosis (Fig. 4).

In order to track the human transplanted cells into the rat renal tissue, we used an anti-human nucleoli antigen antibody. However, we could not track neither the hiPSCs nor the RPC integration into the renal tissue after 45 days of transplantation (Additional figure 1).

## Discussion

The prevalence of CKD has been rapidly increasing, and the treatment of its comorbidities, dietary restriction, such as glycemic restriction, and pressure control do not assure the cessation of the disease progression. Progression of CKD to its end stage leads to treatments that include dialysis and/or renal transplantation [1, 15]. Dialysis is quite complex, reducing the quality of life in patients, whereas renal transplantation, despite being a definitive therapy, is hampered by organ supply and shortcomings associated with immunosuppressive drugs [15]. The blockage and/or regression of CKD progression has been investigated in several studies and clinical trials but they all failed to provide an effective therapeutic approach [16]. For all these reasons, cell therapy has emerged as a promising alternative for CKD treatment [17]. However, it is also noteworthy that only a few studies have currently addressed the use of stem cells in CKD, limiting the possibility of reaching definitive conclusions [3, 4, 18–20].

The main limitations of using iPSCs as cell therapy are due to the accumulation of somatic mutations that can result in tumor formation [21]. In previous studies, we tested the efficacy of iPSCs and BM-MSCs from healthy rats in a 5/6 nephrectomy model and found that cell therapy had the potential to retard the progression of CKD [3]. However, the iPSC generated Wilms’ tumors after transplantation, indicating that the blockage of their proliferative capacity is needed prior to their injection. In the present study, we sought the efficacy of the generated hiPSCs and RPCs in a 5/6 nephrectomy model but differently from other reports, we treated hiPSCs with MMC and differentiated hiPSCs into RPCs, in order to avoid tumor formation [6, 21–26]. MMC is a chemotherapeutic agent capable of suppressing cell proliferation and represents an approach to a safer use of iPSCs in cell therapy. Using a nephrogenic cocktail composed of inducing factors [9, 27–29], we successfully differentiate hiPSCs into RPCs, which presented morphological characteristics and molecular markers typical of the podocyte lineage. Since podocyte injury or depletion may lead to dysfunction and alterations of the glomerular filtration barrier, and given that these cells cannot be naturally replaced after injury [30, 31], we hypothesized that in vitro differentiation of hiPSCs into podocyte progenitor cells could be a potential new approach to improve renal function at the end stage of CKD.

We observed that the body weight loss was reversed in both treated groups, indicating that hiPSCs and RPCs could minimize the impact of CKD on general symptoms, allowing animals to feed regularly, thereby avoiding weight loss, described in CKD [32]. Increased arterial blood pressure levels, another hallmark of CKD, was also partially improved by RPC and hiPSC treatment, suggesting that both cell types may have contributed to ameliorating some pathophysiological mechanisms underlying hypertension in CKD [33, 34].

Interestingly, only hiPSC transplantation significantly improved creatinine clearance in comparison with the CKD and RPC groups. A similar improvement in renal function was also described by some authors using iPSCs and hiPSC-derived renal progenitors [35, 36]. The mechanisms underlying this selective beneficial effect of hiPSCs over the RPC could be due to the release of cytokines and renoprotective factors released by this cell type, as previously reported [35, 37]. Both hiPSCs and RPCs reduced the histological damage observed in CKD. Although it has been proposed that α-SMA may not be a consistent marker of fibrogenic cells [38], the reduction of its positivity in the renal tissue suggests a possible decrease in fibrosis. To corroborate the reduction in the fibrogenic process, we used the IFTA score that also showed a significant decrease in interstitial fibrosis and tubular atrophy in both treated groups.

It has been demonstrated that a reduction in renal fibrosis alone is not sufficient to provide the restoration of kidney function in the absence of nephron regeneration after damage [16]. In our study, we demonstrated that the treatment with hiPSCs in the 5/6 nephrectomy model may have contributed to the improvement in renal function.

In addition, we demonstrated that the hiPSC treatment decreased macrophage CD68+ cells in the renal parenchyma, possibly due to a paracrine effect [35, 37]. Previous studies lend support to the present observation since it has been reported that mouse kidney progenitor-like cells reduce interstitial fibrosis, glomerular sclerosis, recruitment of macrophages and myofibroblasts in the interstitium, and blood pressure levels in the 5/6 nephrectomy model [20].

Some mechanisms have been proposed to explain the beneficial effect of cell therapy on improving renal injury, especially with the use of iPSCs and RPCs [3, 19, 20, 25, 35–37, 39–43]. RPCs can differentiate in vivo by forming renal tubules and acting through pro-proliferative, anti-apoptotic, and anti-inflammatory effects [25], as well as by integrating into the kidney [19], decreasing the level of vascular rarefaction, and preventing endothelial mesenchymal transition [20]. We were not able to localize neither the hiPSCs nor the RPC integration into the renal tissue, after 45 days of transplantation, corroborating the findings described by Chen et al. [20], which have observed only a few RPCs in the injured kidney as early as 28 days after cell transplantation. Thus, our results suggest that both hiPSCs and RPCs may attenuate the CKD damage through paracrine effects. Most importantly, in the present study, we did not observe any tumor formation, neither with the use of hiPSCs nor with RPCs, indicating that the MMC-treated cell approach may be a safer strategy as cell therapy for CKD.

We do acknowledge that a major limitation of our study is the fact that the direct differentiation of hiPSCs into RPCs results in podocyte-like cells in a mature stage, probably limiting their full therapeutic potential. Another limitation is that the mechanisms proposed to explain the beneficial effect of both cell therapies are multifaceted and further studies are required to clarify the exact mechanisms related to the therapeutic effects of hiPSCs and RPCs.

## Conclusions

In conclusion, our study provides evidence that the transplantation of hiPSCs and RPCs in the 5/6 nephrectomized rats may be extrapolated as an effective therapeutic strategy for the end stage of CKD. We also show that by using the MMC-treated hiPSC approach we could reduce CKD damage in the absence of tumor generation. The treatment with both hiPSCs and RPCs reduces weight loss caused by CKD, improves renal function, and reduces fibrosis and glomerulosclerosis. These data represent a promising and safe strategy for CKD treatment, but further studies are necessary to corroborate its therapeutic benefits.

## Supplementary Information

**Additional file 1: Table S1**. Primer list used for qRT-PCR. This table provides a list of all the primers used in this study.

**Additional file 2: Figure S1**. Immunohistochemistry for anti-human nucleoli antigen antibody. Immunohistochemistry for anti-human nucleoli antigen antibody, showing no positive staining for the hiPSC group (A) and the RPC group (B).

## Data Availability

The authors declare that the dataset supporting the conclusions of this study is included within the article and its supplementary information files.

## Abbreviations

CKD: Chronic kidney disease
MMC: Mitomycin C
hiPSCs: Human induced pluripotent stem cells
RPCs: Renal progenitor cells
CCr: Creatinine clearance
BM-MSCs: Bone marrow-derived mesenchymal stem cells
iPSCs: Induced pluripotent stem cells
ESCs: Embryonic stem cells
PBMCs: Peripheral blood mononuclear cells
SCF: Stem cell factor
FBS: Fetal bovine serum
RA: Retinoic acid
EBs: Embryoid bodies
BSA: Bovine serum albumin
cDNA: Complementary DNA
Ct: Threshold cycle
SCr: Serum creatinine
RCCr: Rate of decline of CCr
PT-24h: 24-h proteinuria
HE: Hematoxylin and eosin
IFTA: Interstitial fibrosis and tubular atrophy
IHC: Immunohistochemical
α-SMA: α-Smooth muscle actin
ANOVA: Analysis of variance
eGFR: Estimated glomerular filtration rate
hNPCs: Human nephron progenitor cells
