# Supplementary material for: Therapeutic potential of human induced pluripotent stem cells and renal progenitor cells in experimental chronic kidney disease
Source: Stem Cell Res Ther. 2020 Dec 9;11:530. doi: 10.1186/s13287-020-02060-4 (PMC7727202; doi:10.1186/s13287-020-02060-4)
Supplement: Supplementary file 1 — Additional file 1: Table S1. Primer list used for qRT-PCR. This table provides a list of all the primers used in this study. [file 13287_2020_2060_MOESM1_ESM.docx]

**Additional table 1.** Primer list used for qRT-PCR

| Gene symbol | Gene name | Assay ID |
| --- | --- | --- |
| GAPDH | Glyceraldehyde-3-phosphate dehydrogenase | Hs99999905_m1 |
| BACT | Beta-Actin | Hs01060665_g1 |
| HMBS | Hydroxymethylbilane synthase | Hs00609296_g1 |
| PAX 2 | Paired box 2 | Hs01057416_m1 |
| WT1 | Wilms tumor 1 | Hs01103751_m1 |
| SIX2 | SIX homeobox 2 | Hs00232731_m1 |
| OCT 4 | Octamer-binding transcription factor 4 | Hs04260367_gH |
| NANOG | Nanog homeobox | Hs02387400_g1 |
| SALL 1 | Spalt like transcription factor 1 | Hs01548765_m1 |
| SYNPO | Synaptopodin | Hs00702468_s1 |
| NPHS1 | Nephrin | Hs00190446_m1 |
