# Supplementary material for: Therapeutic potential of human induced pluripotent stem cells and renal progenitor cells in experimental chronic kidney disease
Source: Stem Cell Res Ther. 2020 Dec 9;11:530. doi: 10.1186/s13287-020-02060-4 (PMC7727202; doi:10.1186/s13287-020-02060-4)
Supplement: Supplementary file 2 — Additional file 2: Figure S1. Immunohistochemistry for anti-human nucleoli antigen antibody. Immunohistochemistry for anti-human nucleoli antigen antibody, showing no positive staining for the hiPSC group (A) and the RPC group (B). [file 13287_2020_2060_MOESM2_ESM.docx]

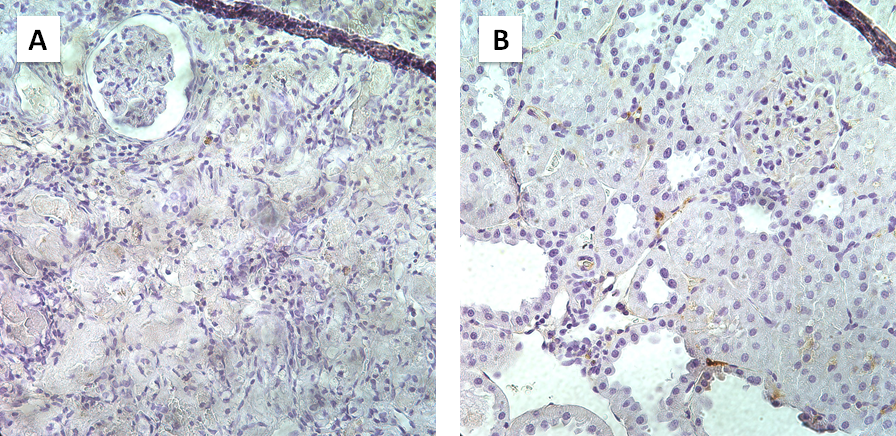


**Additional figure 1.** Immunohistochemistry for anti-human nucleoli antigen antibody. Immunohistochemistry for anti-human nucleoli antigen antibody, showing no positive staining for the hiPSC group (A) and the RPC group (B).
